# From Clinging to Digging: The Postembryonic Skeletal Ontogeny of the Indian Purple Frog, *Nasikabatrachus sahyadrensis* (Anura: Nasikabatrachidae)

**DOI:** 10.1371/journal.pone.0151114

**Published:** 2016-03-30

**Authors:** Gayani Senevirathne, Ashish Thomas, Ryan Kerney, James Hanken, S. D. Biju, Madhava Meegaskumbura

**Affiliations:** 1 Department of Molecular Biology & Biotechnology, Faculty of Science, University of Peradeniya, Peradeniya, Sri Lanka; 2 Systematics Lab, Department of Environmental Studies, University of Delhi, Delhi, India; 3 Department of Biology, Gettysburg College, Gettysburg, Pennsylvania, United States of America; 4 Department of Organismic and Evolutionary Biology and Museum of Comparative Zoology, Harvard University, Cambridge, Massachusetts, United States of America; Ghent University, BELGIUM

## Abstract

The Indian Purple frog, *Nasikabatrachus sahyadrensis*, occupies a basal phylogenetic position among neobatrachian anurans and has a very unusual life history. Tadpoles have a large ventral oral sucker, which they use to cling to rocks in torrents, whereas metamorphs possess adaptations for life underground. The developmental changes that underlie these shifts in habits and habitats, and especially the internal remodeling of the cranial and postcranial skeleton, are unknown. Using a nearly complete metamorphic series from free-living larva to metamorph, we describe the postembryonic skeletal ontogeny of this ancient and unique monotypic lineage. The torrent-dwelling larva possesses a dorsoventrally flattened body and a head with tiny dorsal eyes, robust lower and upper jaw cartilages, well-developed trabecular horns, and a definable gap between the trabecular horns and the tip of the snout. Unlike tadpoles of many other frogs, those of *Nasikabatrachus* retain larval mouthparts into late metamorphic stages. This unusual feature enables the larvae to maintain their clinging habit until near the end of metamorphosis. The subsequent ontogenetic shift from clinging to digging is correlated with rapid morphological changes and behavioral modifications. Metamorphs are equipped with a shortened tibiafibula and ossified prehallical elements, which likely facilitate initial digging using the hind limbs. Subsequently, the frogs may shift to headfirst burrowing by using the wedge-shaped skull, anteriorly positioned pectoral girdle, well-developed humeral crests and spatula-shaped forelimbs. The transition from an aquatic life in torrents to a terrestrial life underground entails dramatic changes in skeletal morphology and function that represent an extreme in metamorphic remodeling. Our analysis enhances the scope for detailed comparative studies across anurans, a group renowned for the diversity of its life history strategies.

## Introduction

*Nasikabatrachus sahyadrensis*, the sole known representative of Nasikabatrachidae, the most recently discovered frog family, is endemic to the Western Ghats of India [1]. Termed “the coelacanth of frogs” in reference to its surprising discovery in 2003 and its distant phylogenetic relationship to most other living frogs [2], *Nasikabatrachus* provides a window into the early history of anurans and gives insight into the evolution of advanced frogs, the neobatrachians. As adults, *Nasikabatrachus* shares a stout body, sturdy limbs and tiny eyes with several burrowing frogs from disparate lineages, and its phylogenetic relationships have been controversial. Its closest living relatives were originally considered to be the Sooglossidae, a family of frogs known only from the Seychelles, an archipelago in the Indian Ocean, with a divergence time of 130 my [1]. There are, however, several morphological dissimilarities between the two families as well as striking differences in their reproductive biology, which might argue against a close relationship. Subsequently, Scott [3] proposed Nasikabatrachidae to be more closely related to the Hemisotidae (comprising a single genus, *Hemisus*—the African purple-nosed frogs) on the basis of external morphology, while Frost *et al*. [4] relegated Nasikabatrachidae a synonym of Sooglossidae. Lack of detailed morphological data led to the further speculation of *Nasikabatrachus* being a hemisotid or a microhylid [5]. Recent phylogenetic studies reaffirm the sister-group relationship between Nasikabatrachidae and Sooglossidae while emphasizing that *Nasikabatrachus* constitutes an independent evolutionary lineage that deserves recognition at the family level [6–8].

*Nasikabatrachus sahyadrensis* has many intriguing features that remain underexplored. The species has large clinging tadpoles that inhabit torrents [9], while metamorphs dig directly into the soil and lead a secretive life underground [1]. Adults are among the largest digging frogs and emerge with the onset of the rainy season to breed in mountain streams [9,10]. Tadpoles use their mouths for feeding, for attachment and for locomotion across submerged boulders in torrents or on the sharp rocky inclines of rheophilic habitats [9,11]. Their oral discs are relatively large, which maximizes both area of attachment and attachment force [9]. While attached, they feed using keratinized teeth.

Adult *Nasikabatrachus* are active diggers that share several specialized external adaptations with other digging frogs, including small yet sturdy heads, small mouths and powerful limbs [1,12]. Adults were not observed—and remained formally undescribed—until recently, in part because of their extremely secretive fossorial habit [1]. In contrast, the very conspicuous tadpoles have been collected by naturalists and utilized by forest-people long before they were scientifically described [13,14]. *Nasikabatrachus* tadpoles begin to dig at Gosner stages 44–45, before metamorphosis is complete and when the residual tail is still being resorbed [9]. They appear to spend the metamorphic climax underground.

Here we describe the metamorphic transformation of the cartilaginous and bony skeleton of *Nasikabatrachus sahyadrensis* based on a nearly complete series of free-living stages from suctorial tadpole to early digging metamorph. We especially highlight the autapomorphies and characters convergent with other digging species, thus contributing to the comparative analysis of anuran skeletal development.

## Materials and Methods

### Ethics statement

This study was conducted with permissions and guidelines from the responsible authorities in the State Forest Departments, Ministry of Environment, Forest and Climate Change, Government of India. The protocol of our collection and research complied with the ethical conditions under the provision of the Wildlife (Protection) Act 1972, Government of India. Specific methods of collection, euthanasia, tissue sampling and fixation followed the guidelines for use of live amphibians and reptiles in field research by the American Society of Ichthyologists and Herpetologists (ASIH) (http://www.asih.org/pubs/herpcoll.html; dated 13 March 2006), and were approved by the internal ethical committee of Department of Environmental Studies, University of Delhi.

### Field surveys and specimen collection

Field collection of tadpoles at Methotti, Kulamaav (09°49’20.83”N, 76°53’29.60”E, 560 m asl) was made by AT and SDB during the monsoon season, between April and September 2008–2013. The tadpoles were randomly sampled from streams to minimize location effects. Following collection, tadpoles were immediately euthanized by immersion in tricaine methanesulfonate (MS-222) and fixed in 10% neutral-buffered formalin. They were subsequently preserved in 70% ethanol.

### External morphology of the tadpole stages

Preserved tadpoles were staged according to Gosner [15]. Tadpoles of *Nasikabatrachus* have a distinctive ventromedial vent-tube with a triangular flap that can obscure the developing hind limb bud. During staging, the flap was deflected with a needle and blue ink applied to assess the limb’s relative development. Tadpoles were measured as in Altig [16] and Altig & McDiarmid [17]. The following external measurements were made to the nearest 0.1 mm by using a digital caliper or a binocular microscope fitted with an ocular micrometer (S1 Table): maximum height of body (BH), maximum length of body (BL), maximum width of body (BW), maximum diameter of eye (ED), internarial distance (NN), naro-pupillar distance (NP), interpupillar distance (PP), rostro-narial distance (RN), distance from tip of snout to opening of spiracle (SS), distance from tip of snout to insertion of upper tail fin (SU), snout-vent length (SVL), total length (TL), distance from vent to tip of tail (VT), tail muscle height (TMH) and tail muscle width (TMW). Specimens are deposited in the Systematics Lab, University of Delhi (SDBDU), under accession numbers SDB 1004–1073.

### Osteology

Osteological preparations and descriptions are based on 69 specimens representing Gosner stages 25–46. All specimens were skinned, eviscerated and differentially stained for bone and cartilage [18], except for one stage-30 specimen that was only stained for cartilage. Initial dehydration in absolute ethanol was followed by cartilage staining using Alcian blue. Specimens were then macerated with trypsin dissolved in 30% aqueous sodium borate, stained for bone using Alizarin red dissolved in 0.5% aqueous KOH, and cleared and ultimately preserved in glycerin. All specimens were scored for bone within one day following staining. When necessary, cleared-and-stained larvae were dissected carefully to observe individual bones and cartilages. A given bone was scored as present based on the presence of calcified (reddish-colored) bone matrix. The presence of calcified endolymph, which also stained red, was recorded separately. Separate ossification indices for cranial and postcranial elements were calculated for each stage: ossification index = number of bones present at a given stage/total number of bones present at stage 46 (S1 Fig). Cleared and stained specimens were photographed with a Nikon SMZ 800 zoom-stereomicroscope with a digital microscope camera (DS-Fi1) and standalone control unit (DL-L3), or with a Canon 60D digital camera with Canon EF 100 mm macro lens or Canon MP-E 65 x1-5 dedicated macro lens. Cartilage terminology follows Cannatella [19] and Hanken & Hall [20]; bone terminology follows Trueb [21], Duellman & Trueb [22], Pugener & Maglia [23,24], Maglia *et al*. [25] and Pugener *et al*. [26].

## Results

A nearly complete ontogenetic series of tadpoles, lacking only Gosner stages 34 and 40, was examined (Table 1). For brevity, only stages 25, 26, 30, 31, 33, 36, 38, 42, 44, 45 and 46 are described below. Description of the neurocranium and the first visceral arch at stage 30 is followed by the morphology of a stage-46 metamorph. Stage 30, which lacks adult nasal cartilages, provides a baseline for evaluating the cartilage remodeling and ossification of subsequent stages. Resolving the morphology of the hyoid skeleton in cleared-and-stained whole mounts of the heavily muscularized larvae of *Nasikabatrachus* proved difficult. Hence, the hyolaryngeal skeleton is described only for stage 46.

**Table 1:** Presence/absence (1/-) of cranial and postcranial bones in *N*. *sahyadrensis* between Gosner stages 25 and 46.

Bones^1^,^2^	Gosner stage																			
	25	26	27	28	29	30	31	32	33	35	36	37	38	39	41	42	43	44	45	46
Frontoparietal	-	1	1	1	1	1	1	1	1	1	1	1	1	1	1	1	1	1	1	1
Parasphenoid	-	-	-	-	-	-	-	-	1	1	1	1	1	1	1	1	1	1	1	1
Exoccipital	-	-	-	-	-	-	1	1	1	1	1	1	1	1	1	1	1	1	1	1
Prootic	-	-	-	-	-	-	-	-	1	1	1	1	1	1	1	1	1	1	1	1
Premaxilla	-	-	-	-	-	-	-	-	-	-	-	-	-	-	-	-	-	-	1	1
Maxilla	-	-	-	-	-	-	-	-	-	-	-	-	-	-	-	-	-	-	1	1
Nasal	-	-	-	-	-	-	-	-	-	-	-	-	1	1	1	1	1	1	1	1
Squamosal	-	-	-	-	-	-	-	-	-	-	-	-	-	-	-	-	-	-	-	1
Angulosplenial	-	-	-	-	-	-	-	-	-	-	-	-	-	-	-	-	-	-	-	1
Dentary	-	-	-	-	-	-	-	-	-	-	-	-	-	-	-	-	-	-	-	1
Pterygoid	-	-	-	-	-	-	-	-	-	-	-	-	-	-	-	-	-	-	-	1
Neural arch I	-	1	1	1	1	1	1	1	1	1	1	1	1	1	1	1	1	1	1	1
Neural arch II	-	-	1	1	1	1	1	1	1	1	1	1	1	1	1	1	1	1	1	1
Neural arch III	-	-	1	1	1	1	1	1	1	1	1	1	1	1	1	1	1	1	1	1
Neural arch IV	-	-	-	1	1	1	1	1	1	1	1	1	1	1	1	1	1	1	1	1
Neural arch V	-	-	-	-	1	1	1	1	1	1	1	1	1	1	1	1	1	1	1	1
Neural arch VI	-	-	-	-	1	1	1	1	1	1	1	1	1	1	1	1	1	1	1	1
Neural arch VII	-	-	-	-	-	1	1	1	1	1	1	1	1	1	1	1	1	1	1	1
Neural arch VIII	-	-	-	-	-	-	-	-	1	1	1	1	1	1	1	1	1	1	1	1
Sacrum	-	-	-	-	-	-	-	-	-	-	1	1	1	1	1	1	1	1	1	1
Centrum I	-	-	-	-	-	1	1	1	1	1	1	1	1	1	1	1	1	1	1	1
Centrum II	-	-	-	-	-	1	1	1	1	1	1	1	1	1	1	1	1	1	1	1
Centrum III	-	-	-	-	-	1	1	1	1	1	1	1	1	1	1	1	1	1	1	1
Centrum IV	-	-	-	-	-	-	1	1	1	1	1	1	1	1	1	1	1	1	1	1
Centrum V	-	-	-	-	-	-	1	1	1	1	1	1	1	1	1	1	1	1	1	1
Centrum VI	-	-	-	-	-	-	1	1	1	1	1	1	1	1	1	1	1	1	1	1
Centrum VII	-	-	-	-	-	-	-	-	1	1	1	1	1	1	1	1	1	1	1	1
Centrum VIII	-	-	-	-	-	-	-	-	1	1	1	1	1	1	1	1	1	1	1	1
Transverse processes I	-	-	-	-	-	-	-	-	-	-	-	-	1	1	1	1	1	1	1	1
Transverse processes II	-	-	-	-	-	-	-	-	-	-	-	-	1	1	1	1	1	1	1	1
Transverse processes III	-	-	-	-	-	-	-	-	-	-	-	-	1	1	1	1	1	1	1	1
Transverse processes IV	-	-	-	-	-	-	-	-	-	-	-	-	1	1	1	1	1	1	1	1
Transverse processes V	-	-	-	-	-	-	-	-	-	-	-	-	-	-	-	-	-	-	1	1
Transverse processes VI	-	-	-	-	-	-	-	-	-	-	-	-	-	-	-	-	-	-	1	1
Transverse processes VII	-	-	-	-	-	-	-	-	-	-	-	-	-	-	-	-	-	-	-	1
Transverse processes VIII	-	-	-	-	-	-	-	-	-	-	-	-	-	-	-	-	-	-	-	1
Sacral diapophysis	-	-	-	-	-	-	-	-	-	-	-	-	1	1	1	1	1	1	1	1
Hypochord	-	-	-	-	-	-	-	-	-	-	1	1	1	1	1	1	1	1	1	1
Coccyx	-	-	-	-	-	-	-	-	-	-	-	-	-	-	-	-	-	-	-	1
Urostyle	-	-	-	-	-	-	-	-	-	-	-	-	-	-	-	-	-	-	-	1
Scapula	-	-	-	-	-	-	-	-	-	-	1	1	1	1	1	1	1	1	1	1
Humerus	-	-	-	-	-	-	-	-	-	-	1	1	1	1	1	1	1	1	1	1
Ulna	-	-	-	-	-	-	-	-	-	-	1	1	1	1	1	1	1	1	1	1
Radius	-	-	-	-	-	-	-	-	-	-	1	1	1	1	1	1	1	1	1	1
Cleithrum	-	-	-	-	-	-	-	-	-	-	-	-	1	1	1	1	1	1	1	1
Clavicle	-	-	-	-	-	-	-	-	-	-	-	-	1	1	1	1	1	1	1	1
Coracoid	-	-	-	-	-	-	-	-	-	-	-	-	1	1	1	1	1	1	1	1
Metacarpal II	-	-	-	-	-	-	-	-	-	-	-	-	1	1	1	1	1	1	1	1
Metacarpal III	-	-	-	-	-	-	-	-	-	-	-	-	1	1	1	1	1	1	1	1
Metacarpal IV	-	-	-	-	-	-	-	-	-	-	-	-	1	1	1	1	1	1	1	1
Metacarpal V	-	-	-	-	-	-	-	-	-	-	-	-	1	1	1	1	1	1	1	1
FL Phalange digit II-1	-	-	-	-	-	-	-	-	-	-	-	-	-	-	1	1	1	1	1	1
FL Distal phalange digit II	-	-	-	-	-	-	-	-	-	-	-	-	-	-	-	-	1	1	1	1
FL Phalange digit III-1	-	-	-	-	-	-	-	-	-	-	-	-	1	1	1	1	1	1	1	1
FL Distal phalange digit III	-	-	-	-	-	-	-	-	-	-	-	-	-	-	-	-	1	1	1	1
FL Phalange digit IV-1	-	-	-	-	-	-	-	-	-	-	-	-	1	1	1	1	1	1	1	1
FL Phalange digit IV-2	-	-	-	-	-	-	-	-	-	-	-	-	-	-	1	1	1	1	1	1
FL Distal phalange digit IV	-	-	-	-	-	-	-	-	-	-	-	-	-	-	-	-	1	1	1	1
FL Phalange digit V-1	-	-	-	-	-	-	-	-	-	-	-	-	-	-	-	-	1	1	1	1
FL Phalange digit V-2	-	-	-	-	-	-	-	-	-	-	-	-	-	-	1	1	1	1	1	1
FL Distal phalange digit V	-	-	-	-	-	-	-	-	-	-	-	-	-	-	-	-	1	1	1	1
Femur	-	-	-	-	-	-	-	-	-	-	-	-	1	1	1	1	1	1	1	1
Tibia	-	-	-	-	-	-	-	-	-	-	-	-	1	1	1	1	1	1	1	1
Fibula	-	-	-	-	-	-	-	-	-	-	-	-	1	1	1	1	1	1	1	1
Fibulare	-	-	-	-	-	-	-	-	-	-	-	-	1	1	1	1	1	1	1	1
Tibiale	-	-	-	-	-	-	-	-	-	-	-	-	1	1	1	1	1	1	1	1
Ilium	-	-	-	-	-	-	-	-	-	-	1	1	1	1	1	1	1	1	1	1
Ischium	-	-	-	-	-	-	-	-	-	-	-	-	-	-	-	-	1	1	1	1
Metatarsal I	-	-	-	-	-	-	-	-	-	-	-	-	1	1	1	1	1	1	1	1
Metatarsal II	-	-	-	-	-	-	-	-	-	-	-	-	1	1	1	1	1	1	1	1
Metatarsal III	-	-	-	-	-	-	-	-	-	-	-	-	1	1	1	1	1	1	1	1
Metatarsal IV	-	-	-	-	-	-	-	-	-	-	-	-	1	1	1	1	1	1	1	1
Metatarsal V	-	-	-	-	-	-	-	-	-	-	-	-	-	-	-	-	1	1	1	1
HL Phalange digit I-1	-	-	-	-	-	-	-	-	-	-	-	-	1	1	1	1	1	1	1	1
HL Distal phalange digit I	-	-	-	-	-	-	-	-	-	-	-	-	-	-	-	-	1	1	1	1
HL Phalange digit II-1	-	-	-	-	-	-	-	-	-	-	-	-	1	1	1	1	1	1	1	1
HL Distal phalange digit II	-	-	-	-	-	-	-	-	-	-	-	-	-	-	-	-	1	1	1	1
HL Phalange digit III-1	-	-	-	-	-	-	-	-	-	-	-	-	1	1	1	1	1	1	1	1
HL Phalange digit III-2	-	-	-	-	-	-	-	-	-	-	-	-	-	-	1	1	1	1	1	1
HL Distal phalange digit III	-	-	-	-	-	-	-	-	-	-	-	-	-	-	-	-	1	1	1	1
HL Phalange digit IV-1	-	-	-	-	-	-	-	-	-	-	-	-	1	1	1	1	1	1	1	1
HL Phalange digit IV-2	-	-	-	-	-	-	-	-	-	-	-	-	-	-	1	1	1	1	1	1
HL Phalange digit IV-3	-	-	-	-	-	-	-	-	-	-	-	-	-	-	1	1	1	1	1	1
HL Distal phalange digit IV	-	-	-	-	-	-	-	-	-	-	-	-	-	-	-	-	1	1	1	1
HL Phalange digit V-1	-	-	-	-	-	-	-	-	-	-	-	-	-	-	-	-	1	1	1	1
HL Phalange digit V-2	-	-	-	-	-	-	-	-	-	-	-	-	-	-	-	-	1	1	1	1
HL Distal phalange digit V-4	-	-	-	-	-	-	-	-	-	-	-	-	-	-	-	-	1	1	1	1
Distal prehallical element 1	-	-	-	-	-	-	-	-	-	-	-	-	-	-	-	-	-	-	1	1
Distal prehallical element 2	-	-	-	-	-	-	-	-	-	-	-	-	-	-	-	-	-	-	1	1

^1^Abbreviations: FL, forelimb; HL, hind limb.

^2^Septomaxilla, vomers, quadratojugals, columella, mentomeckelians, palatines, sphenethmoid, and teeth are not present. Radiale, ulnare, carpals, prepollical elements, sternum, omosternum, tarsal-2, and element Y are cartilaginous even at stage 46.

### Larval neurocranium and the first visceral arch (stage 30)

**Fig 1:**
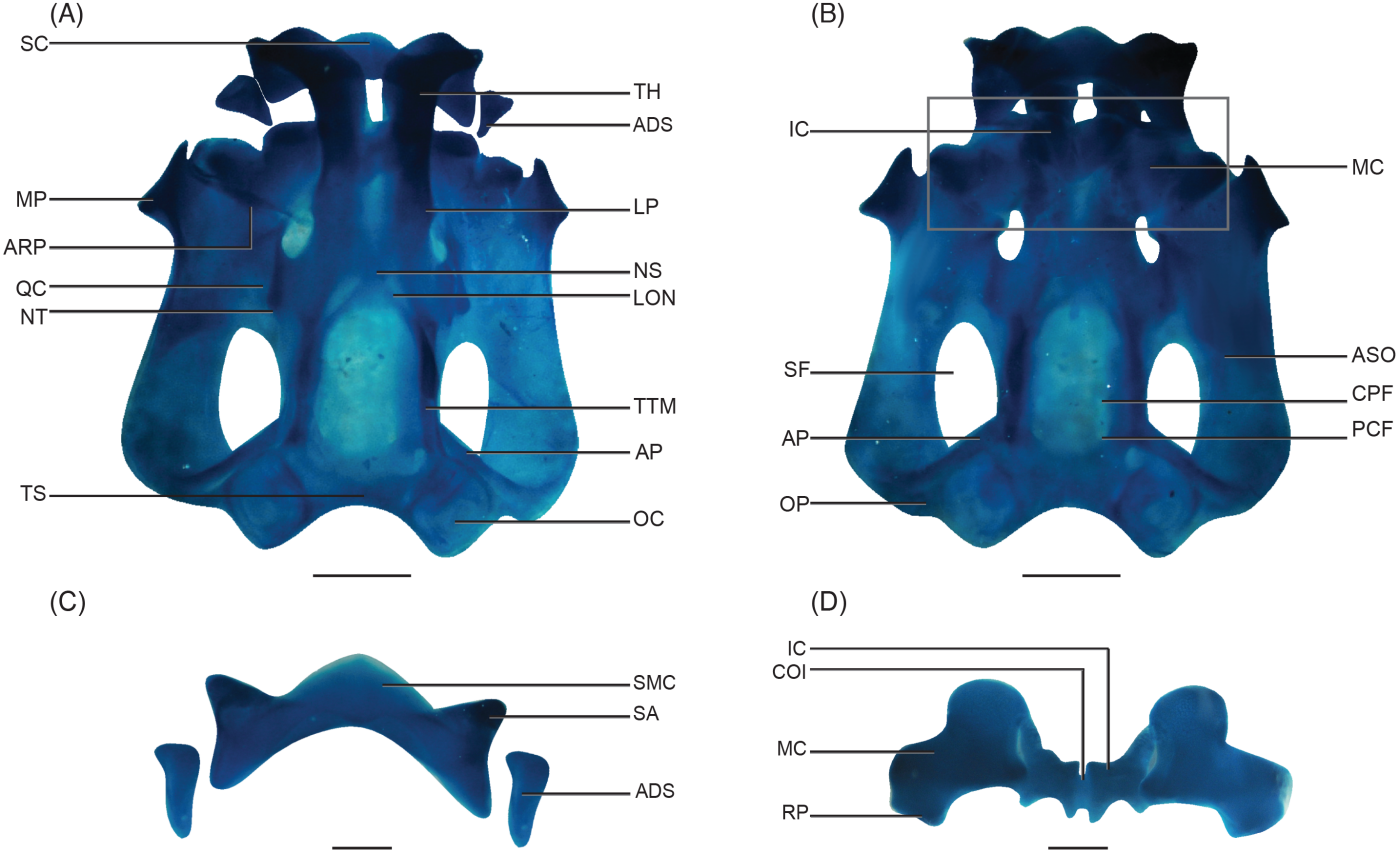
Alcian blue-stained neurocranium and the first visceral arch of *Nasikabatrachus sahyadrensis* at stage 30. (A) Dorsal view. (B) Ventral view. (C) Upper jaw. (D) Lower jaw. Abbreviations: ADS, adrostral cartilage; AP, ascending process; ARP, articular process; ASO, arcus subocularis; COI, commissura intramandibularis; CPF, craniopalatine foramen; IC, infrarostral cartilage; LON, lamina orbitonasalis; LP, lateral process; MC, Meckel’s cartilage; MP, muscular process; NS, nasal septum; NT, nasal tectum; OC, otic capsule; OP, otic process; PCF, primary carotid foramen; QC, quadratocranial commissure; RP, retroarticular process; SA, suprarostral ala; SC, suprarostral cartilage; SF, subocular fenestra; SMC, suprarostral medial corpus; TH, trabecular horns; TS, synotic tectum; TTM, taenia tecti marginalis. Scale bars: 1 mm.

Description of the larval neurocranium is based on four stage-30 tadpoles (Fig 1A–1D). The average width of the cranium equals 99% of its length (*N =* 4); the greatest width is attained at the level of the muscular processes. Trabecular horns extend from the ethmoid plate and are relatively long—46% of chondrocranial length (Fig 1A). The horns are uniform in width but diverge anteriorly, resulting in a V-shape with an angle of 74° with the ethmoid plate. A lateral process arises from the lateral margin of each trabecular horn near its base. Anteriorly, each trabecular horn curves to articulate with lateral alae and central corpus of the suprarostral (upper jaw) cartilage on the same side.

The single suprarostral cartilage is oriented dorsoventrally. It comprises a medial corpus bordered laterally by two well-defined, triangular alae (Fig 1A–1C). Dorsal and ventral margins of the suprarostral are complete; anterolateral margins articulate with the posteriorly curved anterior ends of the trabecular horns via synovial joints. A pair of well-developed adrostral cartilages are present dorsal, lateral and posterior to the posterior margin of the suprarostral alae (Fig 1A and 1C). Paired, wedge-shaped infrarostral cartilages are joined medially by a cartilaginous commissura intramandibularis, and each infrarostral articulates anterolaterally with an L-shaped Meckel’s cartilage (Fig 1D). Ramaswami [27] describes from serial sections a second connective-tissue attachment between infrarostral and Meckel’s cartilages of a given side. We could not discern this connective tissue in whole mounts, but in other species the component, which also is termed commissura intramandibularis, is often discernible only in sections [28]. Each Meckel’s cartilage articulates posteriorly with the articular process of the palatoquadrate and is positioned obliquely to the longitudinal axis of the neurocranium.

The paired palatoquadrate cartilages are located lateral to the braincase and are concave in dorsal view. The anterior margin of each articular process (“pars articularis quadrati” [26]) overlaps the posterior margin of Meckel’s cartilage. The arcus subocularis (“suborbital cartilage” [26]) is roughly spherical. The palatoquadrate is expanded at its anterolateral margin, giving rise to a reduced muscular process and quadratocranial commissure. The apex of the muscular process lies anterior to the quadratocranial commissure. The anterior portion of the arcus subocularis gives rise to the hyoquadrate process ventrally, which articulates with the ceratohyal cartilage of the hyobranchial skeleton (not shown). The ascending process articulates with the lateral braincase wall slightly dorsal to the oculomotor foramen. A flat, plate-like otic process is present at the posterolateral corner of the palatoquadrate. The muscular process has a ligamentous attachment to the processus antorbitalis of the lamina orbitonasalis.

A cartilaginous nasal septum separates the paired nasal capsules and merges posteriorly with the tectum nasi, which roofs the nasal capsules. Tectum nasi arise laterally as rounded projections that are located dorsally to the anterior quadratocranial commissure. The lamina orbitonasalis comprises a small triangular projection medial to the anterior quadratocranial commissure. The frontoparietal fenestra is undivided and bordered by the taenia tecti marginalis. The fenestra is 36% of the total length of the chondrocranium and widest at the anterior margin of the otic capsules. Taenia tecti transversalis and taenia tecti medialis are absent.

### Metamorphosed froglet (stage 46)

#### Cranium

**Fig 2:**
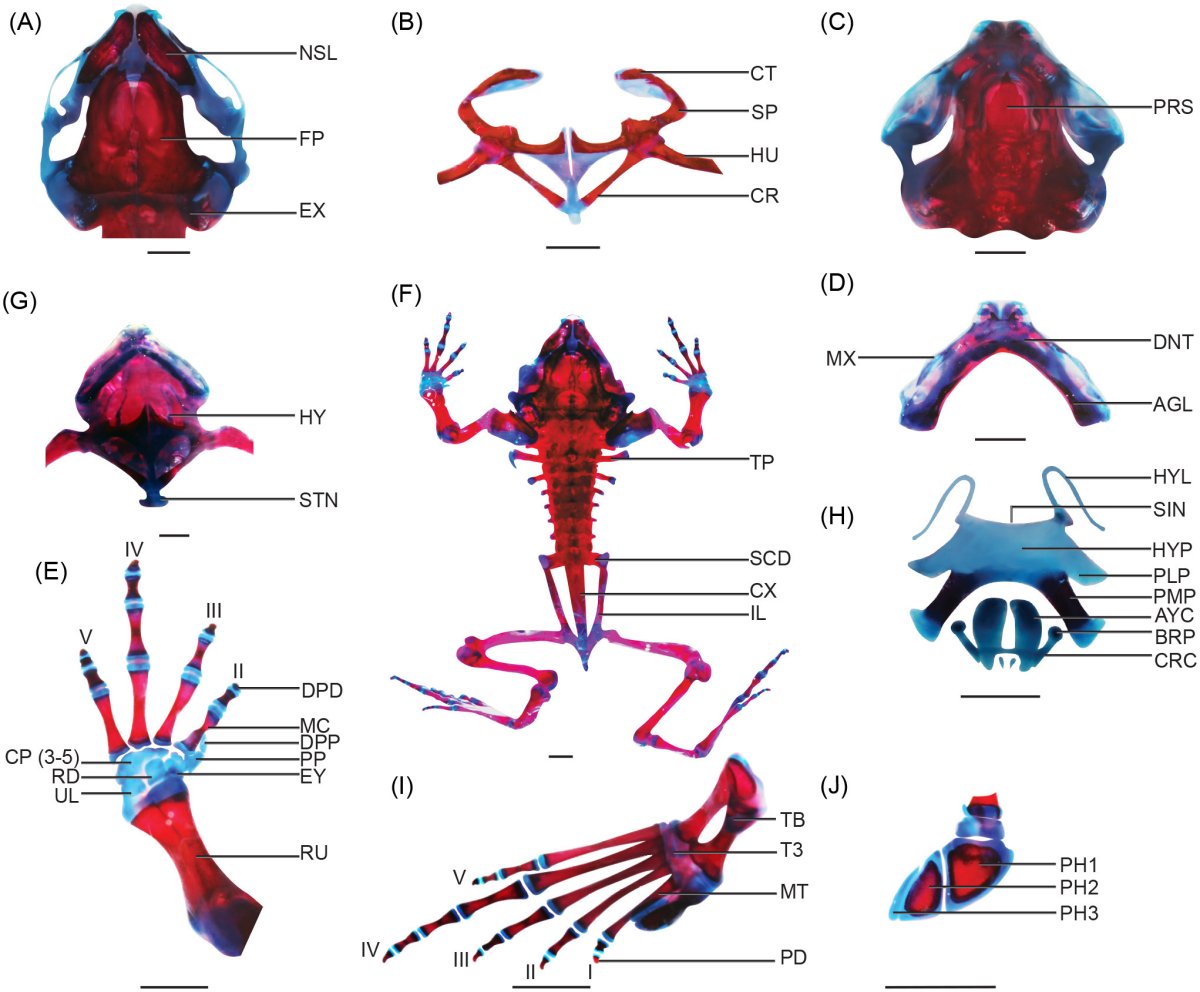
Skeleton of a metamorphosed froglet of *N*. *sahyadrensis*, stage 46. (A) Cranium. (B) Pectoral girdle. (C) Cranium. (D) Lower and upper jaw. (E) Right forelimb. (F) Fully stained juvenile. (G) Cranium and pectoral girdle. (H) Hyoid plate and laryngeal cartilages. (I) Right hind limb. (J) Right prehallux. All are ventral views except C and I (dorsal) and J (lateral). Abbreviations: AGL, angulosplenial; AYC, arytenoid cartilage; BRP, bronchial process; CP (3–5), fused carpals 3–5; CR, coracoid; CRC, cricoid cartilage; CT, cleithrum; CX, coccyx; DNT, dentary; DPD, distal phalange digit; DPP, distal prepollex; EX, exoccipital; EY, element Y; FP, frontoparietal; HU, humerus; HYL, hyale; HYP, hyoid plate; IL, ilium; MC, metacarpals; MT, metatarsals; MX, maxilla; NSL, nasal; PD, phalange digit; PH, prehallux; PH1, distal prehallux 1; PH2, distal prehallux 2; PH3, distal prehallux 3; PLP, posterolateral process; PMP, posteromedial process; PP, prepollex; PRS, parasphenoid; RD, radiale; RU, radioulna; SCD, sacral diapophyses; SIN, hyoglossal sinus; SP, scapula; STN, sternum; TB, tibiale; TP, transverse process; T3, tarsal 3; UL, ulnare. Cartilage is blue, bone is red. Scale bars: 1 mm.

Digging metamorphs. The skull is narrow anteriorly and wide posteriorly; it is widest at the anterior margins of the otic capsules (Fig 2A). The palatoquadrate cartilage is opposite the otic capsules and oriented dorsoventrally (Fig 2A). The ossified neurocranium contains paired prootics and exoccipitals; the sphenethmoid is absent (Fig 2A and 2C).

Prootics and exoccipitals are paired endochondral bones. Prootics constitute anteromedial and anterolateral portions of the otic capsules, which are only slightly ossified. Posterolateral portions of the otic capsules, posterior margins of the neurocranium and margins of the foramen magnum are formed by paired exoccipitals, which are distinct from the prootics.

Frontoparietals are large dorsal bones that extend over two thirds of the skull’s length and nearly two thirds of its width. Their anterior ends approach the posterior margins of the nasals but do not articulate with them. Posterolaterally, the frontoparietals overlie the prootics and cover one fourth of the otic capsules in dorsal view. Frontoparietals are distinct from one another except for a slight area of fusion along the midline (Fig 2A).

Paired nasal bones invest the skull dorsally. They are well developed and densely ossified (Fig 2A). Trapezoid-shaped nasals completely cover the olfactory capsules posteriorly. They are separated from one another medially by a thin nasal septum and do not articulate with either the maxillae or the premaxillae. In one specimen, paired nasals nearly overlap posteriorly with the anterior margins of the frontoparietals.

The median, unpaired parasphenoid is inverted, T-shaped, azygous and forms the ventral dermatocranium (Fig 2C). Anteriorly, the cultriform process invests the nasal septum. It extends to the posterior margins of the nasals in ventral view. Two small lateral processes extend from the cultriform process at the level of the posterior margin of the orbits. Rectangular alae have blunt distal ends that extend laterally, perpendicular to the cultriform process, and invest the ventral two thirds of the otic capsules. Posterior margins of the alae converge medially to form an inverted, U-shaped depression.

**Fig 3:**
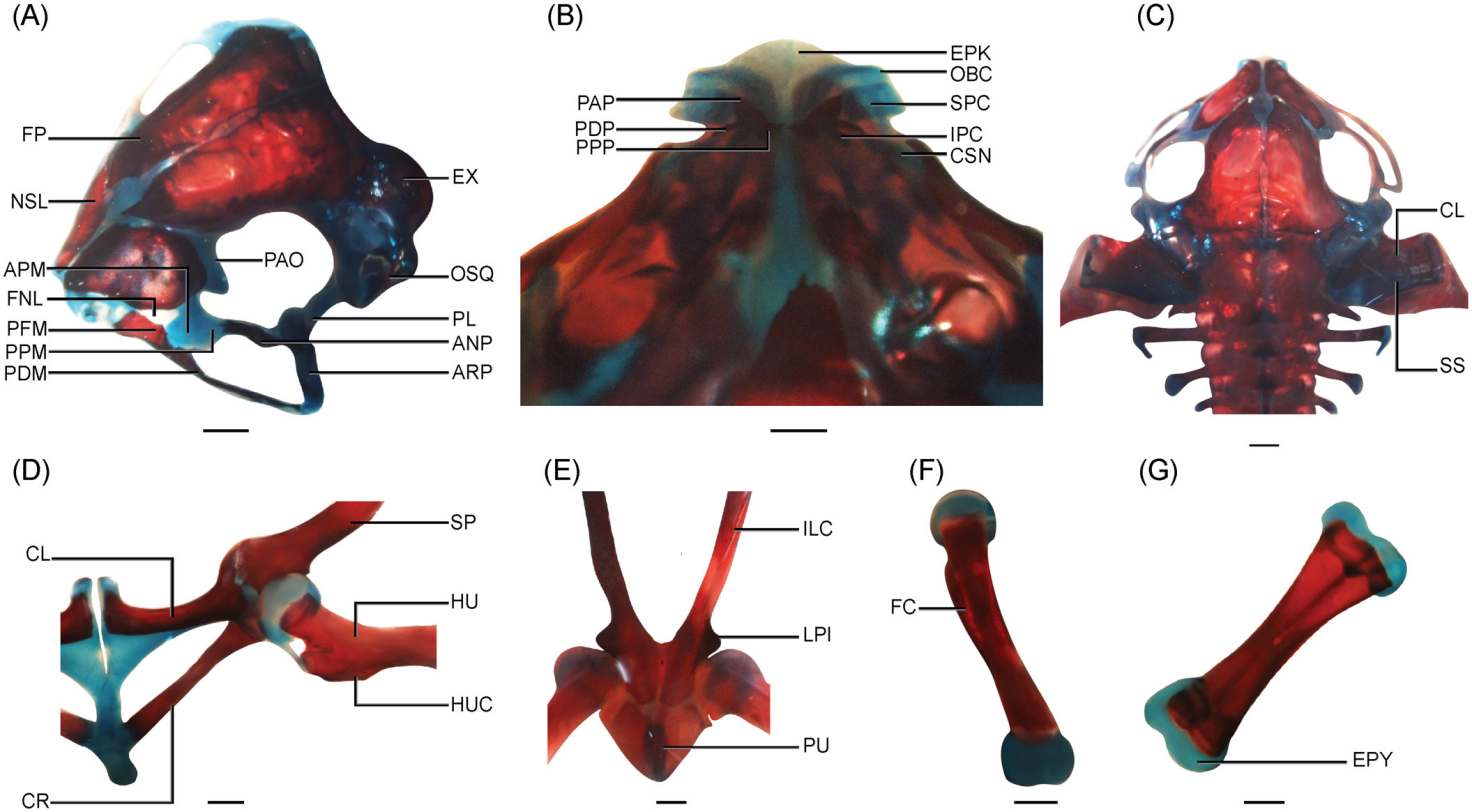
Close-ups of *N*. *sahyadrensis*, stage 46. (A) Lateral oblique view of the skull. (B) Ventral view of the anterior skull. (C) Attachment of the pectoral girdle to the axial skeleton. (D) Articulation of the humeral head with the glenoid fossa. (E) Fusion of ilia. (F) Long cylindrical femur. (G) Tibiafibulare. Abbreviations: ANP, anterior ramus of pterygoid; APM, anterior maxillary process; ARP, articular process; CL, clavicle; CR, coracoid; CSN, crista subnasalis; EPK, epidermal knob; EPY, cartilaginous epiphysis; EX, exoccipital; FC, femoral crest; FNL, fenestra nasolateralis; FP, frontoparietal; HU, humerus; HUC, humeral crest; ILC, ilial crest; IPC, inferior prenasal cartilage; LPI, lateral process of ilium; NSL, nasal; OBC, oblique cartilage; OSQ, otic process of squamosal; PAO, planum antorbitale; PAP, pars alaris of premaxilla; PDM, pars dentalis of maxilla; PDP, pars dentalis of premaxilla; PFM, pars facialis of maxilla; PL, degenerating palatoquadrate; PPM, posterior maxillary process; PPP, pars palatina premaxilla; PU, pubis; SP, scapula; SPC, superior prenasal cartilage; SS, suprascapula. Scale bars: 1 mm.

Pterygoids are paired, Y-shaped bones that begin to ossify along the pterygoid process of the palatoquadrate (Fig 3A). Each pterygoid possesses three rami—anterior, posterior and medial—which are recognizable but not well developed. Laterally, the posterior ramus is positioned anterodorsally and almost perpendicular to the maxilla and has a pointed end. The anterior ramus is the most developed. It extends anteromedially from where posterior and medial rami arise and terminates in a square end. It does not yet articulate with the maxilla. The medial ramus, pointed and horn shaped, is poorly developed compared to anterior and posterior rami and does not articulate with the otic capsule.

Paired squamosals invest the cranium posterolaterally in one of three specimens (Fig 3A). When fully formed, ventral, zygomatic and otic rami give these dermal bones an inverted T-shape. However, only the otic ramus is observed at this stage, along the ventral side of the otic process. The auditory apparatus is absent.

#### Maxillary arcade

Three paired bones—maxilla, quadratojugal and premaxilla—along with cartilaginous inferior prenasals, crista subnasalis and planum antorbitale, constitute the maxillary arcade.

Paired premaxillae are narrowly separated from one another and do not articulate with the maxillae (Fig 3B). The dentary process (pars dentalis) is triangular and edentate; the pars palatina is poorly developed (Fig 3A and 3B). A well-chondrified crista subnasalis occurs between the dentary process and the pars facialis of the maxilla (Fig 3A and 3B).

Paired, edentate maxillae are the longest components of the arcade. The pars facialis is well developed; it extends to the posterolateral margin of the nasal but does not articulate with it (Fig 3A). The pars dentalis extends anteriorly towards but remains separate from the pars dentalis of the premaxillae. Posteriorly, maxillae extend to the level of the anterior margins of the orbit capsules. The pars palatine is not yet present.

Paired quadratojugal bones are not present at this stage (metamorphosed froglet).

#### Mandible

Meckel’s and infrarostral cartilages are thin and rod-like (Fig 2D). Elongate Meckel’s cartilages have reoriented with respect to the infrarostrals and form a shallow arch in ventral view. Angulosplenial bones invest the posterior margins of Meckel’s cartilage and extend anteriorly to the planum antorbitale. Posteriorly, the angulosplenial bone reaches the articular joint of the lower jaw; it bears a triangular coronoid process. Paired dentary bones invest the anterolateral margin of Meckel’s cartilage and do not articulate with any other bone or cartilage at this stage. Mentomeckelian bones have not yet formed.

#### Hyoid skeleton

The thin, flat hyoid plate, along with paired hyale, posteromedial processes and posterolateral processes, constitute the hyoid skeleton. These, together with the laryngeal apparatus (below), comprise the hyolaryngeal complex (Fig 2H). Posteromedial and posterolateral processes are equally wide, but the posteromedial process is longer. The posteromedial process contains a bony shaft, which ends in a cartilaginous epiphysis. Anterolateral processes are absent. The hyoglossal sinus is shallow and bounded laterally by a pair of thin hyale. A foreshortened hyoid plate is located just beneath the pectoral girdle in ventral view (Fig 2J).

### Laryngeal apparatus

A partially developed laryngeal apparatus is situated between the posteromedial processes of the hyoid skeleton. It comprises a pair of hemi-spherical arytenoid cartilages and a pair of incomplete cricoid cartilages. Arytenoids are broader than the cricoid cartilages. The anterior-most end of each cricoid cartilage expands to form an irregular-shaped bronchial process.

#### Vertebral column

**Fig 4:**
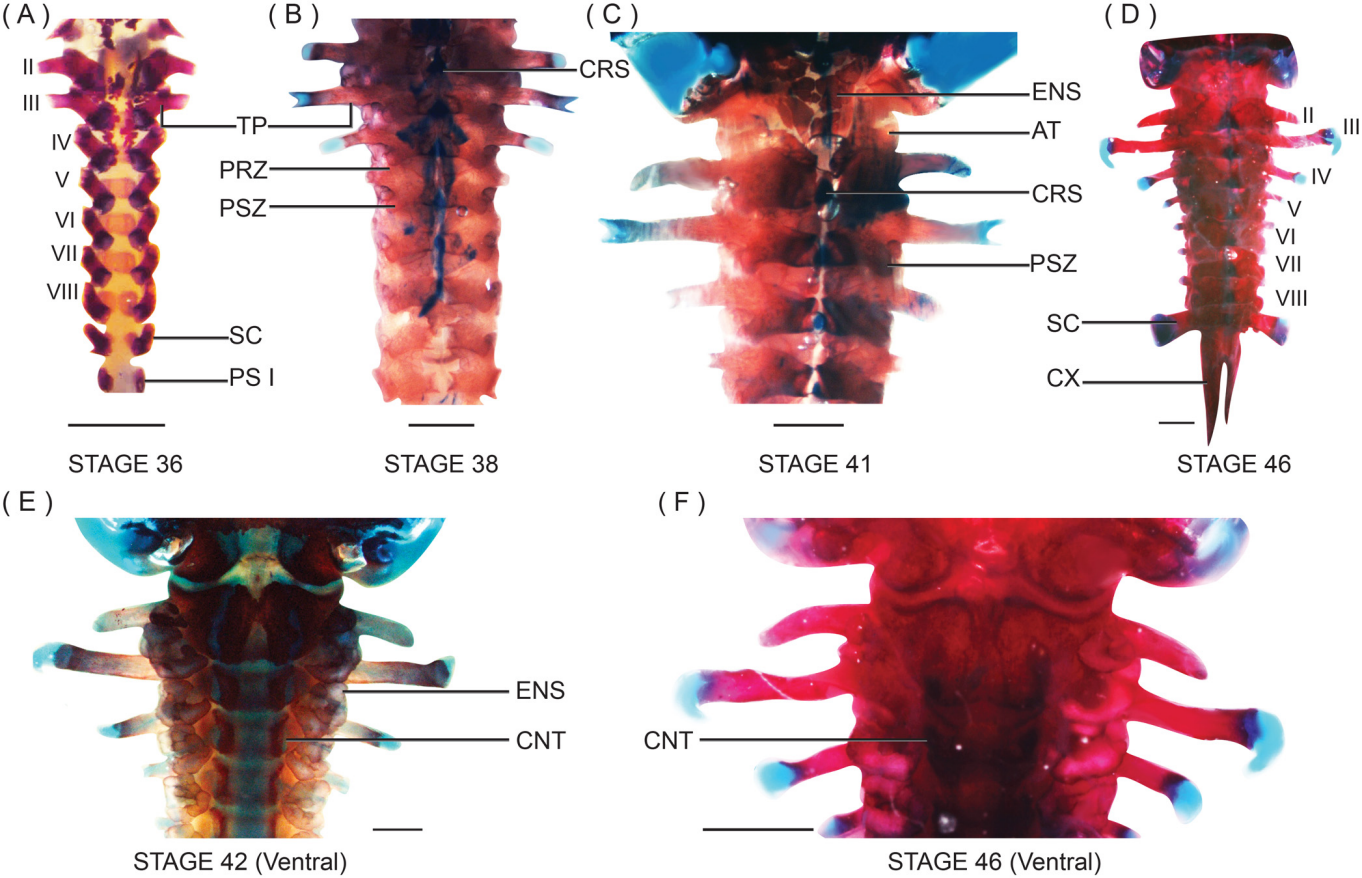
Development of the axial skeleton and endolymphatic sacs in *N*. *sahyadrensis*. (A) Axial skeleton at stage 36. (B) Axial skeleton at stage 38. (C) Close-up of the axial skeleton at stage 41. (D) Fully developed axial skeleton at stage 46. (E) Axial skeleton at stage 42. (F) Close-up of the cervical vertebra at stage 46. A–D are dorsal views; E and F are ventral views. Abbreviations: AT, atlas; CNT, ossified centrum; CRS, cartilaginous strip; CX, coccyx; ENS, endolymphatic sacs; PRZ, prezygapophyses; PS I, first postsacral vertebra; PSZ, postzygapophyses; SC, sacrum; TP, transverse process; II–VIII, vertebrae 2–8. Scale bars: 1 mm.

The vertebral column comprises presacral, sacral and postsacral regions. The fused atlas + second presacral vertebra (also known as the cervical) along with six additional presacral vertebrae constitute the presacrals (Fig 2F). The fused centrum of the cervical is larger than the other centra (Fig 4D and 4F). The cervical bears a pair of cervical cotyles with pointed ossified surfaces, which articulate laterally with occipital cotyles. Presacrals possess procoelous centra. However, the cervical has a wing-like anterior margin and a shallow medial depression (Fig 4F). Imbricate neural arches of the presacrals are well developed. All possess prezygapophyses anteriorly and postzygapophyses posteriorly except the cervical, which only has postzygapophyses. Neural arches adjoin medially via a cartilaginous body. The cervical bears two transverse processes on each side, which extend posterolaterally. Presacral III bears the longest and widest transverse processes, which are inclined posteriorly (Fig 4D). All transverse processes are ossified proximally but terminate in cartilage distally. The transverse process of presacral IV is nearly half the size of presacral III. The length of the transverse processes decreases posteriorly, beginning with presacral III. The postzygapophysis of presacral VIII articulates with the prezygapophysis of the sacrum. The sacrum bears a pair of dilated, posteriorly inclined zygapophyses, which articulate with the ilium. The sacrum articulates with the coccyx posteriorly. The coccyx is fused with the hypochord anteriorly (which is completely ossified except for the cartilaginous terminus) and forms the urostyle.

#### Pectoral girdle

Stage-46 metamorphs have arcifero-firmisternal pectoral girdles: epicoracoid cartilages are fused, whereas interclavicle regions of the well-developed procoracoid cartilages are not ([21]; Fig 2B). The caudal portion of the pectoral girdle lacks horn-like extensions. The procoracoids articulate with the scapulae laterally and are posteromedially contiguous with the epicoracoids (Fig 2B). Clavicles are well ossified; they invest anterior regions of the procoracoid cartilages but do not fuse with scapular ossifications. Coracoid bones invest the epicoracoid cartilages completely and extend to their medial articulation. Epicoracoids are narrow and lack horns; they are fused with the cartilaginous sternum posteriorly. The omosternum is absent (Figs 2B and 3D). The scapulae are small compared to the clavicles (Fig 3D). Lateral ends of the coracoids are slightly larger than medial parts. The suprascapula is attached to the scapula and endochondral ossification of the suprascapula can be seen along its posterior margin. The cleithrum is ossified along the anterior margin of the suprascapula and invests almost half of the cartilaginous suprascapula. Anterior margins of the cleithra overlap the posterolateral margins of the otic capsules (Fig 3C).

#### Pelvic girdle

The pelvic girdle consists of paired ilia, ischia and pubes (Fig 3E). Ilia are fused posteromedially; they bear processes on the lateral margins. Each ilial crest is distinguishable along the posterior margin (Fig 3E). The posterolateral margin of the ilium articulates with the anterolateral margin of the ischium. The pubis is cartilaginous in the metamorphosed froglet.

#### Forelimb

The proximal end of the humerus articulates with the posterior half of the glenoid fossa within the pectoral girdle. The humeral head is subtended by a prominent crest (Fig 3D). Radioulnae are fused medially but remain separate at their epiphyses. The manus comprises five carpals, two prepollical elements, four metacarpals and four digits (Fig 2E). The phalangeal formula is 2-2-3-3 (Fig 2E). All elements are ossified except the carpals and prepollical elements. The ulnare and radiale are the same size, whereas element Y is slightly smaller. Carpal 2, the smallest carpal, is distal to element Y, whereas fused carpals 3, 4 and 5 are distal to the ulnare and radiale. Two cartilaginous prepollical elements are located lateral to carpal 2. The distal prepollex has a pointed apex and is smaller than the prepollex. Elongate, cylindrical metacarpals articulate with the carpals proximally. Metacarpal IV is the longest, followed by III, V and II. Distal phalanges of digits II–IV are small and curved inwards.

#### Hind limb

The femur is well ossified, including a crest along its anterior margin (Fig 3F). Its distal epiphysis is slightly larger than the proximal acetabular epiphysis. Each tibiafibula is slightly shorter than the femur (Fig 3G) and has a large proximal and a small distal head. Tibia and fibula are fused but can be differentiated by a medial sulcus (Fig 2I). Tibiale and fibulare are nearly half the length of femur. The fibulare is slightly longer than the tibiale. They are separated from one another at midlength but fused medially at their proximal and distal cartilaginous epiphyses. The distal cartilaginous head of the tibiale articulates with prehallical elements. The prehallux consists of four elements (Fig 2J). The centers of distal prehallical elements 1 and 2 are ossified (margins are cartilaginous), whereas the prehallux and distal prehallical element 3 are completely cartilaginous. Tarsals 1, 3 and 4–5 are cartilaginous. The five metatarsals are long, slender, cylindrical bones with cartilaginous proximal and distal ends. Metatarsal IV is the longest, followed by III, V, II and I. The phalangeal formula is 2-2-3-4-3 (Fig 2I). Distally, most terminal phalanges are smaller and curved inwards.

### Development of the bony skeleton during metamorphosis

#### Stage 25

**Fig 5:**
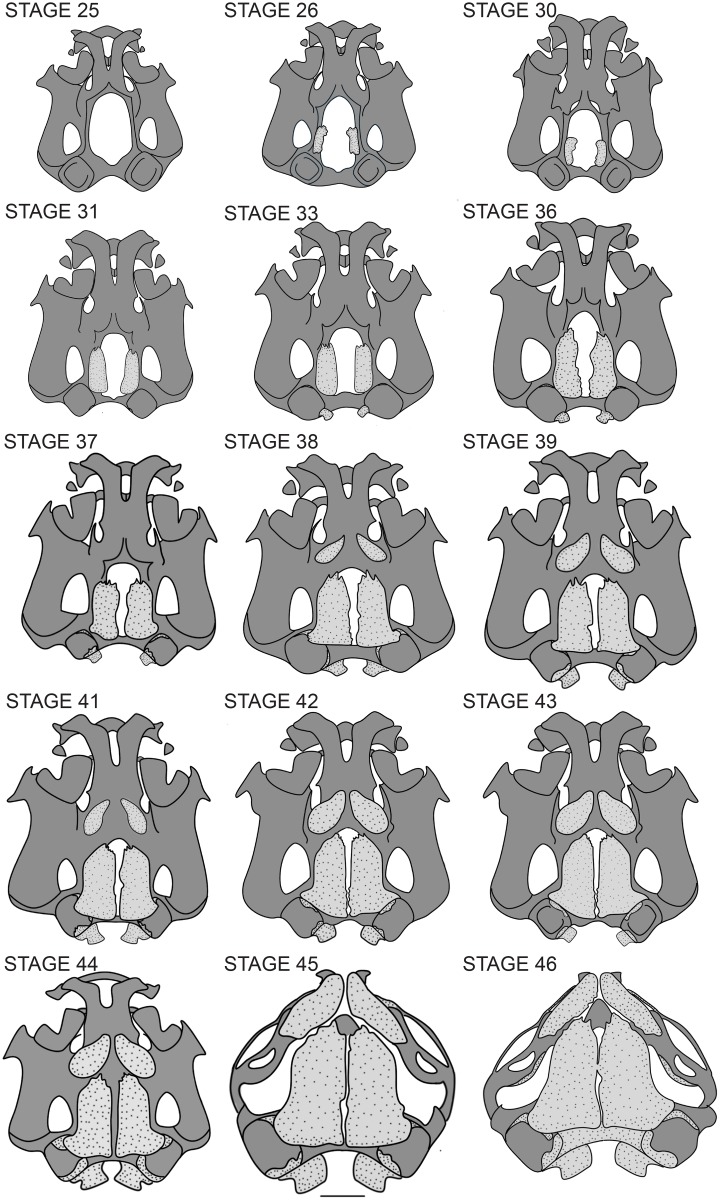
Development of the chondrocranium into a highly ossified cranium in *N*. *sahyadrensis*. Paired frontoparietals, exoccipitals and parasphenoids are the first three bones to form; nasals and frontoparietals completely cover the well-developed crania at later stages.

In life, these free-living larvae were found near the egg-laying site attached to the substrate by their suctorial mouthparts. Maximum skull width is 91% of maximum length; trabecular horns are relatively long, ca. 40% of chondrocranial length (Fig 5). Patches of calcified endolymph (“lime sacs” [22]) are visible near the inner anterolateral margin of each auditory capsule, but bone is absent (Table 1).

#### Stage 26

The suctorial larvae have tiny limb buds. In life, they make an initial move into the stream habitat, where they orally attach to the substratum. Cranial ossification begins. Initial ossification of the paired frontoparietal bones occurs along the taenia tecti marginalis (Fig 5). These ossification centers will extend both anteroposteriorly and medially and eventually form the cranial roof. The nasal septum is poorly developed. Calcified endolymphatic sacs extend along the anteroposterior margins of the auditory capsules and also along the extradural space of the vertebral column. Postcranial osteogenesis is also beginning, as revealed by faint ossifications within the neural arch pedicles of the atlas vertebra.

#### Stage 30

Clinging tadpoles. Frontoparietal ossifications extend to the lateral margin of each otic capsule, covering one third of the undivided frontoparietal fenestra (Fig 5). Endolymphatic sacs are larger. Faint ossifications are present at the bases of the neural arch pedicles of presacral vertebrae I–VII. The base of the pedicle of the cervical vertebra (fused atlas + presacral II) is twice as large as those of other vertebrae. Cartilaginous transverse processes also are present on the cervical vertebra and presacral III; each has pointed distal termini and is oriented anteriorly.

#### Stage 31

Frontoparietal and vertebral ossifications are more extensive (Fig 5). Paired exoccipital bones have begun to form along the posterior margins of the foramen magnum. Calcified endolymphatic sacs are larger and more numerous and extend posteriorly from the auditory capsules. Paired ossification centers are visible within each centrum of the cervical vertebra and of presacrals III, IV, V, VI and VII. Transverse processes have formed on the cervical vertebra.

#### Stage 33

Paired frontoparietals extend further anteroposteriorly and medially but are not fused (Fig 5). Prootic and parasphenoid bones appear for the first time. Faint ossifications of the paired prootics outline the anterior margins of the otic capsules. The cultriform process of the median, unpaired parasphenoid is visible as a single ossification center along the ventral midline of the neurocranium. Thin exoccipital ossifications extend along the occipital arches, forming an occipital condyle on either side. The corresponding atlantal condyles have also begun to ossify. Transverse processes of the cervical vertebra and presacral III develop laterally. Rudimentary paired ossifications are visible along the cartilaginous centra of presacral VIII. Neural arch laminae of all presacral vertebrae are partially ossified.

#### Stage 36

Ossification is further advanced (Fig 5). Frontoparietals reach the anterior margin of the orbits anteriorly and the midpoint of the otic capsules posteriorly. Medially, they now occupy approximately half of the original frontoparietal fontanelle. Prootics extend laterally to form the anterior walls of the auditory capsules; each occipital arch now has an occipital condyle with a slightly concave posterior surface. Ventrally, the parasphenoid has attained a triradiate shape by developing a pointed cultriform process anteriorly and two lateral alae. Endolymphatic sacs are now visible within the neurocranium and extend further posteriorly along the vertebral column; they are concentrated along the presacral neural arch laminae III, IV and V. Presacral vertebrae have well-ossified neural arches and chondrified prezygapophyses and postzygapophyses. Ossification has begun in the neural arch lamina of the sacrum and neural arch pedicles of postsacral vertebra I. The hypochord is visible as a thin medial ossification posterior to the sacrum and postsacral vertebra I. Ossification is beginning in the appendicular skeleton.

Each half of the pectoral girdle comprises cartilaginous primordia of the procoracoid, coracoid and scapula + suprascapula (first seen at stage 35). Initially, the scapula comprises a diaphyseal ossification within the cartilaginous primordia of the fused scapula + suprascapula. The humerus ossifies initially along the midpoint of its cartilaginous shaft. Radius and ulna are initially unfused; ossification is initiated as two faint centers along their cartilaginous shafts. Diapophyses of the cartilaginous primordia of the ilia are the first bones to ossify within the pelvic girdle. The femur is beginning to ossify within its cartilaginous shaft concurrently with osteogenesis of the cartilaginous primordia of all long bones, metatarsals (I–V) and phalanges (Table 1).

#### Stage 38

In life, larvae still cling to the substratum via their mouthparts, which are intact and well developed. The chondrocranium is wider than long. Paired frontoparietals have expanded anteroposteriorly and medially (Fig 5). Medial separation of these bones is ca. 6% of the width of the chondrocranium. Paired, crescent-shaped nasal bones are beginning to ossify and invest the anterior roof of the nasal capsule. They do not articulate medially. Prootics have grown laterally and posteriorly and now constitute up one fourth of the otic capsules. Ventrally, the parasphenoid has grown anteriorly and laterally. It extends anteriorly to the rostral margin of the frontoparietal fenestra and laterally invests nearly half of each otic capsule. Each laterally extending ala terminates in a bifurcated tip. Exoccipitals are well ossified along the jugular foramen but are not fused medially.

Presacral neural arches are fused medially via a cartilaginous strip (Fig 3B). Transverse processes of the cervical vertebra and presacrals III and IV are well developed; lateral ends are expanded slightly (Fig 4B). The sacrum bears two round diapophyses oriented perpendicular to the vertebral column. The ossified sacral diapophyses expand laterally and terminate in cartilage. The coccyx comprises a pair of elongate ossification centers posterior to the first postsacral vertebra and is truncated posteriorly. The two ossification centers do not articulate with the hypochord or with one another. Paired ossification centers within the cartilaginous centrum of the cervical vertebra are partly fused medially. Endolymphatic sacs extend along the vertebral column caudal to the sacrum.

**Fig 6:**
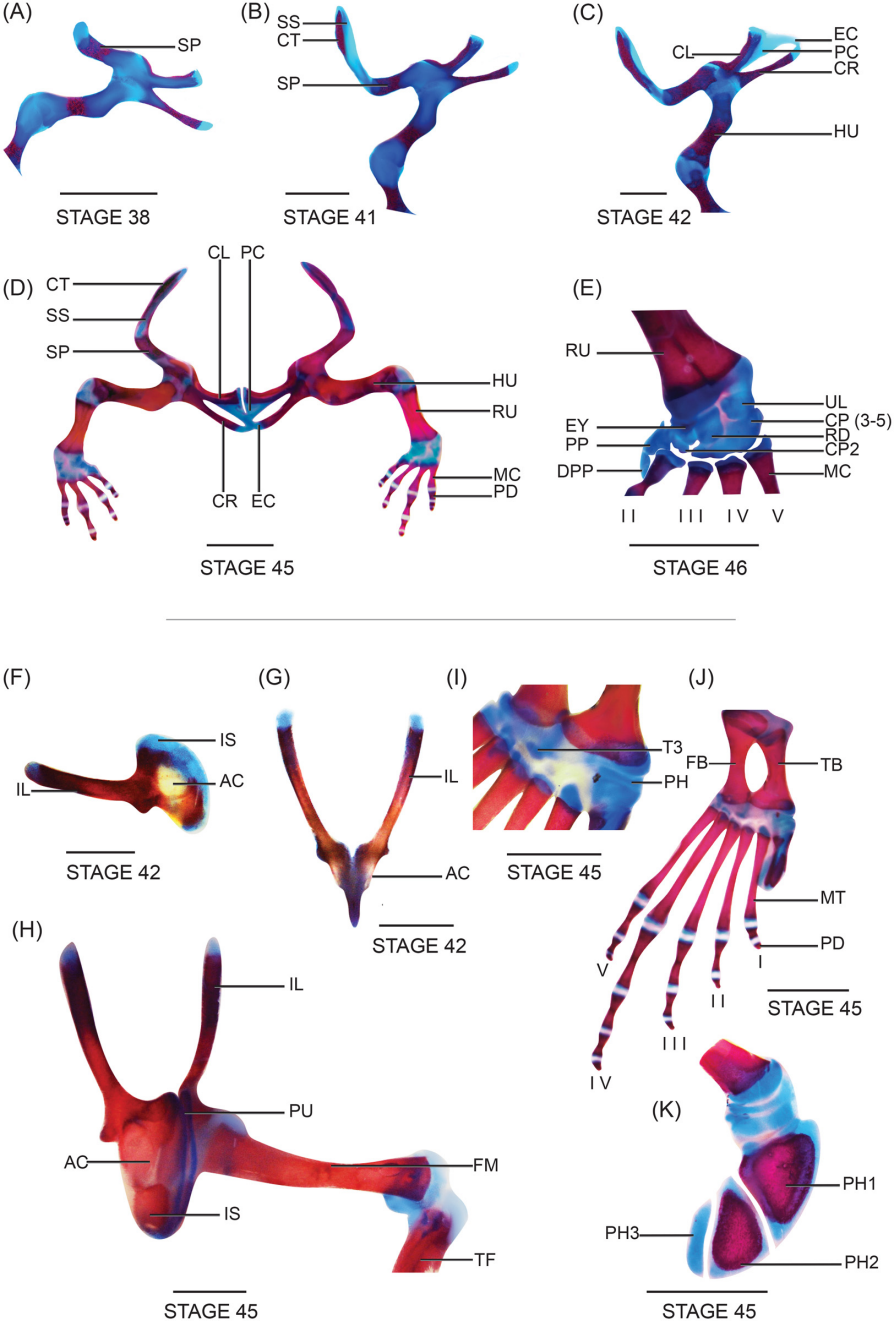
Development of the appendicular skeleton in *N*. *sahyadrensis*. (A) Right side of the pectoral girdle at stage 38. (B) Right side of the pectoral girdle at stage 41. (C) Right side of the pectoral girdle at stage 42. Note the cartilaginous epicoracoid, bridging clavicle and coracoid. (D) Forelimbs attached to the pectoral girdle via proximal end of humeri, stage 45. (E) Manus at stage 46. (F) Lateral view of the pelvis at stage 42. (G) Dorsal view of the pelvis at stage 42. (H) Dorsolateral view of the pelvic girdle at stage 45. (I) Dorsal view of tarsals at stage 45. (J) Dorsal view of hind limb at stage 45. (K) Lateral view of prehallux at stage 45. Abbreviations: AC, acetabulum; CL, clavicle; CP2, carpal 2; CP (3–5), fused carpals 3–5; CR, coracoid; CT, cleithrum; DPP, distal prepollex; EC, epicoracoid cartilage; EY, element Y; FB, fibulare; FM, femur; HU, humerus; IL, ilium; IS, ischium; MC, metacarpals; MT, metatarsals; PC, procoracoid cartilage; PD, phalange digits; PH, prehallux; PH1, distal prehallux 1; PH2, distal prehallux 2; PH3, distal prehallux 3; PP, prepollex; PU, pubis; RD, radiale; RU, radioulna; SP, scapula; SS, suprascapula; TB, tibiale; TF, tibiafibulare; T3, tarsal 3; UL, ulnare. Scale bars: 1 mm.

Ossification of the appendicular skeleton begins before metamorphic climax. Scapular ossifications extend along the scapular cartilage. The paired cleithra, clavicles and coracoids are beginning to ossify (Fig 6A). The clavicle extends from the medial margin of the suprascapula cartilage along the anterior border of the procoracoid cartilage. The cylindrical coracoid extends from the diaphysis of the epicoracoid cartilage. Developing forelimbs are visible beneath the operculum. Radius and ulna are longer and fused medially. The cartilaginous primordium of the ulnare is visible, but other carpals are not. A single cartilaginous prepollical element is present, adjacent to the second digit. Metacarpals II–V and proximal phalanges of digits III and IV ossify as medial centers along their corresponding cylindrical cartilages. Ilia are longer, but neither the ischium nor the pubis is visible. Most of the long bones within the hind limb are visible. The femur extends along the diaphysis of its cartilaginous primordium. Medial ossifications are visible within the cartilaginous primordia of the remaining long bones of the hind limb—tibiafibulare, tibiale and fibulare. Tibia and fibula are fused medially but free at their proximal and distal ends. Cartilaginous primordia of tarsals and the prehallux are present. Metatarsals and proximal phalanges of digits I–V are ossified medially.

#### Stage 42

Early metamorphs, which have well-developed fore- and hind limbs, have moved towards the margins of the fast-flowing streams. The chondrocranium is now wider than long and partially covered by bone. Larval jaw cartilages remain intact (Fig 5). Medial separation of the paired frontoparietals is 1% of the width of the chondrocranium. Paired nasals remain separate but overlie the nasal capsules posteriorly (Fig 5). Prootics extend anterolaterally along the otic capsule margins. In ventral view, the parasphenoid reaches the anterior margin of the frontoparietal fenestrae. Exoccipitals are well developed and include posterior margins of the otic capsules, but the margins of the foramen magnum are not completely ossified. Occipital condyles and well-developed cervical cotyles articulate via cartilaginous facets (Fig 4E).

The vertebral column is only slightly advanced from earlier stages. Small transverse processes have developed on presacral vertebrae V, VI, VII and VIII, but they are barely visible in comparison to the well-developed processes of presacral III (Fig 4E). Postsacral vertebra I is fused to the anterior portion of the coccyx. Endolymphatic sacs extend outwards from the extradural spaces of the vertebral column. They are especially visible in the areas where the transverse processes arise.

Pectoral and pelvic girdles are well developed; their component bones have increased in length and breadth. Cartilaginous procoracoids and epicoracoids articulate medially to form an arch, which separates the two halves of the pectoral girdle (Fig 6C). Cleithrum extends along the anterior margin of each epicoracoid and overlies two thirds of the suprascapular cartilage. Slight ossifications of the suprascapula appear on the posterolateral margin of the suprascapular cartilage. Ossification of the clavicle, coracoid and scapula is more extensive. Ilia of the pelvic girdle are well ossified but do not articulate medially; each displays an ilial crest along its posterior margin (Fig 6F and 6G). Ischia have begun to ossify along their outermost margins. The pubis is well chondrified.

The forelimb, hind limb, manus and pes are similar to those at stage 46 (metamorphosed froglet) except for ossification of prehallical elements. The center of the distal prehallical element 1 is ossified (with cartilaginous margins), whereas the prehallux and distal prehallical elements 2 and 3 are still cartilaginous.

#### Stage 44

Metamorphs have moved towards the banks of slow-flowing drainages. Few conspicuous changes of the developing cranium have occurred since stage 42, apart from continued growth of bones. Trabecular horns are beginning to erode; their distal ends are now located ventrolaterally. Originally square tips of the trabecular horns are more rounded. Suprarostral cartilages are degrading medially. Meckel’s and infrarostral cartilages are still distinguishable, but Meckel’s cartilage is beginning to elongate. The muscular process of the palatoquadrate is located further posteriorly; the articular process has broadened anteriorly. Appendicular and axial skeletons resemble those at stage 42 except for distal prehallical element 2, which is beginning to ossify.

#### Stage 45

Metamorphs burrow into soil near stream banks and remain underground through metamorphic climax. Extensive change has occurred since stage 44. Trabecular horns have degenerated. The palatoquadrate is extensively remodeled and has regressed posteriorly. The muscular process has eroded. A synchondrosis now unites the anterior portion of the quadratocranial commissure and the posterior maxillary process with the pterygoid process of the palatoquadrate. Meckel’s cartilage has elongated considerably and retains its articulation with the palatoquadrate. Frontoparietals and nasals are larger; they remain separate from one another and from other bones, except for frontoparietal overlap with the prootics laterally. Ossification of paired maxillary bones is beginning. The cartilaginous anterior maxillary process extends from the planum antorbitale and articulates with the pars facialis of the maxilla, while the posterior maxillary process extends posteroventrally to articulate with the cartilaginous pterygoid process of the palatoquadrate. Paired, edentate premaxillary bones assume an inverted “T” shape. Each has an alary process and slightly developed pars dentalis. Rod-like superior prenasal cartilages end abruptly near the anterior margins of the alary processes. The cartilaginous plate between the tectum nasi and well-developed oblique cartilages is eroding, forming the fenestra nasolateralis (Fig 3A). Inferior prenasal cartilages approach the posterior part of the alary process. Postcranial skeletons resemble those of stage 46 (Fig 6D and 6H–6K).

## Discussion

The unique post-hatching ontogeny of *Nasikabatrachus* includes an abrupt metamorphosis from a torrent-dwelling, clinging larva to a fossorial froglet. Indeed, the shift to an underground microhabitat is completed even before metamorphic climax. We document several characters and ontogenetic patterns that provide a structural framework for this dramatic transformation. They also reveal character convergence with larval and adult frogs of disparate lineages that have similar habits and occupy similar habitats.

Generalized, stream-dwelling tadpoles use their tails to swim and their suctorial mouths to browse on suspended and stationary food [17,22,29]. In a torrent habitat, however, free-floating food is accessible to only the most agile swimmers. Consequently, torrent-dwelling tadpoles typically feed on stationary food sources such as aufwuchs associated with submerged boulders or algae growing on exposed boulders in splash zones. To feed on these firmly attached food sources, torrent-inhabiting tadpoles typically have the oral disc modified to serve as a ventral sucker, which is supported by a specialized cartilaginous skeleton.

Suctorial tadpoles have evolved in several disparate neobatrachian lineages in response to the similar ecological challenges posed by fast-flowing streams. Stream-dwelling *Nasikabatrachus sahyadrensis* are characterized by dorsoventrally flattened bodies, heavy muscularized tails, a medial vent-tube with a triangular flap, robust serrated jaw sheaths and suctorial mouthparts with marginal and submarginal papillae [9]. The suctorial larvae (stages 25–43) of *N*. *sahyadrensis* share this condition with *Ascaphus truei* [30–32], *Heleophryne* spp. [27], *Hyla claresignata* [33], *Litoria* and *Nyctimystes* [34], and *Amolopus* and *Ansonia* [35].

Ramaswami [27] identified the torrent-dwelling tadpoles initially reported by Annandale [36], now known to be *N*. *sahyadrensis*, as“cystignathids.” By comparing these tadpoles with those of *Heleophryne*, he speculated that torrent-dwelling larvae might resemble one another externally but not internally. He reported trabecular horns absent in *Heleophryne* but present in cystignathids; the processus ascendens absent in *Heleophryne* but present in cystignathids; and operculum development during metamorphosis in *Heleophryne* but during premetamorphosis in cystignathids. In contrast, Haas & Richards [34] conclude that suctorial tadpoles show convergence of both external and internal features; these include expanded trabecular horns, robust infrarostrals, fusion of the suprarostrals, a robust palatoquadrate and an enlarged ventral process of the palatoquadrate for articulartion with the orbitohyoideus muscle. Similarly, we show that the chondrocranium of *N*. *sahyadrensis* shares several conspicuous features with those of other lineages.

In *Nasikabatrachus*, a well-developed larval chondrocranium persists from stage 25 until stage 44, when the skull undergoes a dramatic metamorphosis. Trabecular horns are narrow, elongate and possess anteriorly truncated tips; they diverge anteriorly to support the well-developed upper jaws (suprarostral cartilage). Attachment of the trabecular horns to the suprarostral via both its central corpus and lateral alae—a synapomorphy in bufonids, dendrobatids and hylids [37]—is also seen in *Nasikabatrachus*. Similarly, a pair of well-developed adrostral cartilages adjacent to the posterior and lateral ends of the suprarostral also is a conspicuous feature of many other suctorial tadpoles, including *Litoria nannotis*, *L*. *rheocola* and *L*. *dayi* [34], *Rhinella quechua* [38], and *Heleophryne*, *Megophrys* and *Spea* [39]. It is likely that this feature evolved independently in *Nasikabatrachus*, a phylogenetically basal lineage. Haas & Richards [34] propose that, in *Litoria*, each adrostral functions as a “pushing rod” which transmits force to the suprarostral. Additional study is needed to see if the same functional mechanism applies to the convergently evolved adrostral cartilages in *N*. *sahyadrensis* and other disparate lineages of neobatrachians that inhabit fast-flowing streams.

The lower jaws are robust in *N*. *sahyadrensis*, *Hyla claresignata* [33], *Litoria* and *Nyctimystes* [34]. Such altered proportions of underlying jaw cartilages and trabecular horns may serve to buttress the enlarged mouthparts and suctorial discs of torrent-dwelling tadpoles [35]. Similarly, the adherent oral discs aid in the “hitching” movement of larvae over the substrate [34]. A robust palatoquadrate with broad anterior quadratocranial commissure and well-developed posterior articular process is present in *Hyla claresignat*a [33], *Litoria* and *Nyctimystes* [34], as well as in *Nasikabatrachus*.

**Fig 7:**
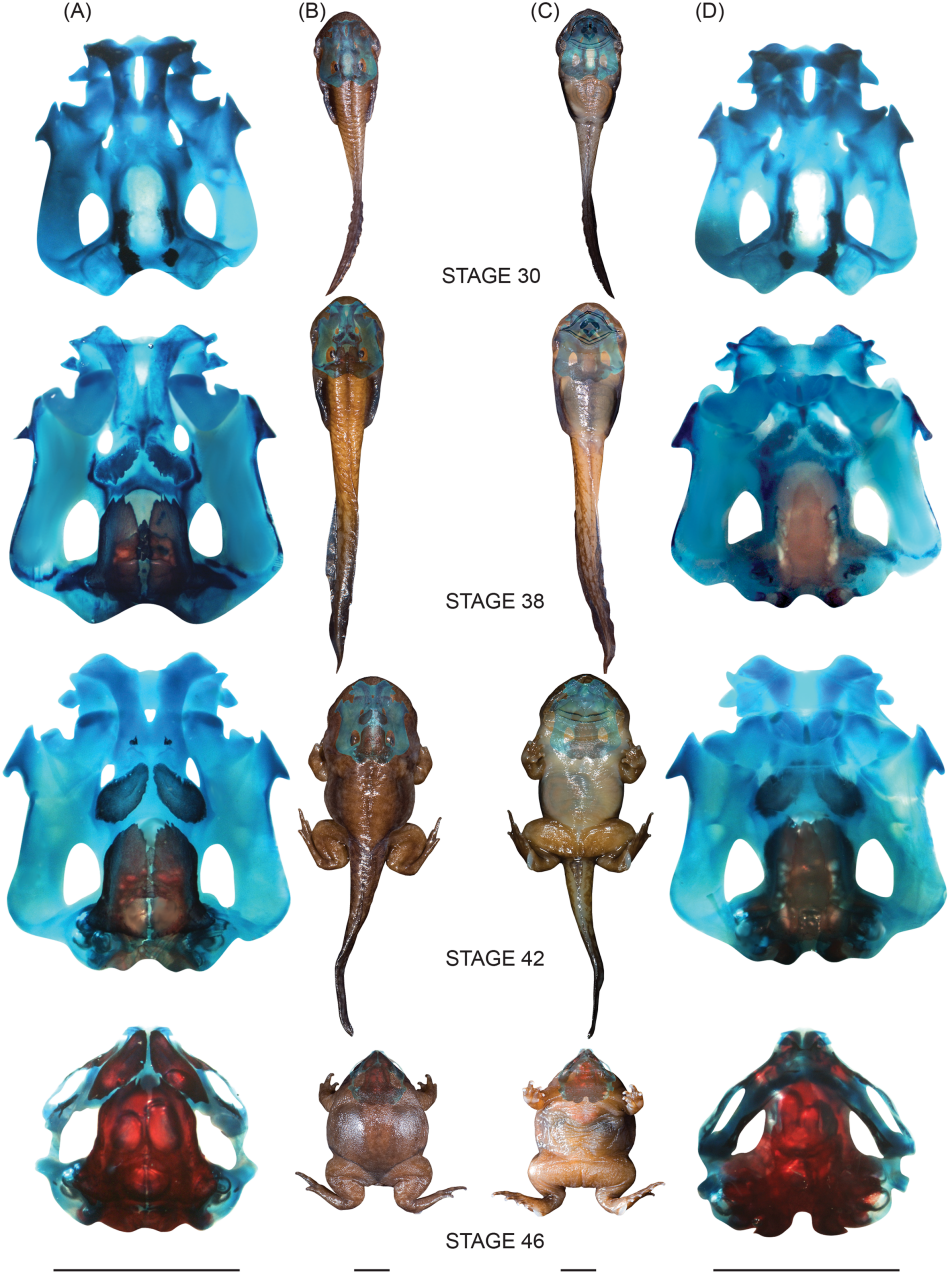
Internal and external morphological modifications at stages 30, 38, 42 and 46 seen in dorsal (A, B) and ventral (C, D) views.

Suctorial larvae have shovel-shaped, depressed bodies, which help them press against or even attach to submerged rocks and boulders and resist strong currents [34]. Small dorsal eyes, which facilitate aerial vision when moving along rocks, are also characteristic of suctorial tadpoles [35], as is a considerable gap between the rostral end of the trabecular horns and the tip of the snout, which is often filled with a semi-gelatinous tissue and may function as a shock absorber [33]. These features are also present in *Nasikabatrachus* (Fig 7).

The transition from a torrent-dwelling, clinging larva to a metamorphosed adult presents a “broken or greatly damaged” ontogenetic sequence whereby larval mouthparts are retained into late developmental stages when compared to the metamorphosis of a typical tadpole, in which loss of larval mouthparts is congruent with absorption of the tail [35]. As the tadpoles of *N*. *sahyadrensis* age and move towards the edges of streams, dramatic modifications of chondrocrania occur rapidly and osteogenesis of postcranial and cranial elements coincide with the new requirements of their burrowing lifestyle. This metamorphic pattern resembles those of other torrent-dwellers such as *Amolops*, *Ansonia* [35] and *Ascaphus truei* [40,41].

A “hitching” action, whereby tadpoles of *Nasikabatrachus* in splash zones scrape algae from rocks covered by a thin film of water, is observed during stages 26–44 [9–14]. The same behavior is reported for *Ansonia longidigita* and *Amolops orphnocnemis* [35], *Adenomus kandianus* [42], *Bokermannohyla claresignata* [33], *Ascaphus truei* [30,31], *Ghatophryne ornata* [9] and *Rhinella quechua* [38].

Metamorphosis is a vulnerable period for most anuran larvae due to the substantial developmental rearrangements and associated changes in habits and habitats that occur during that time [43,44]. In that light, persisting within the splash zone, a microhabitat that offers protection from both strong stream currents and aquatic predation, until the well-developed tail is absorbed may be of considerable survival value [35]. In *Nasikabatrachus*, rapid loss of the specialized larval mouthparts and suctorial oral disc coincides with the onset of fossoriality at stage 45. Metamorphs spend little time moving between these two specialized and protective habitats.

Cartilaginous lower and upper jaws are retained into late metamorphic stages (43–44; Fig 5). There also is a lag in the elongation of Meckel’s cartilage and corresponding increase in the size of the gape (Fig 5). By stage 44 all postcranial elements are well ossified in comparison to cranial elements (Table 1 and S1 Fig). The shift to a fossorial life involves additional internal and external transformations that enhance digging and feeding abilities in this new “zone of safety.”

Recently metamorphosed *Nasikabatrachus sahyadrensis* (stages 45–46) migrate towards the banks of streams where they begin to burrow. They possess stout bodies accommodating adaptations for burrowing. These include large frontoparietals and nasals, which are extensively ossified and almost completely cover the cranium dorsally; both features are considered plesiomorphic for burrowing anurans [21]. As is true for nearly all species of digging frogs, *Nasikabatrachus* digs predominantly with its hind feet (the remaining species dig headfirst [45]). The frogs push against the substrate with their hind feet, often concentrating the force in the vicinity of the metatarsal tubercle. In *N*. *sahyadrensis*, well-ossified prehallical elements of the strong metatarsal tubercles (Figs 2J and 6K, Table 1) presumably are used as spades to shift soil [45,46]. The shortened tibiafibula (19% of SVL) enhances the force of the metatarsal tubercle during digging [45] and may facilitate quadrupedal locomotion (walking) underground [45,47]. Emerson [45] speculated that well-developed humeral crests and robust humeri enable attachment of broad pectoral and forelimb muscles. Both features are present in burrowing forms such as *Hemisus* and *Glyphoglossus* [45] and in *N*. *sahyadrensis*.

The pectoral girdle of *Nasikabatrachus* is located at the rostral-most end of the trunk. Indeed, it partially overlaps the prootics and exoccipitals at the caudal end of the skull (Figs 2F and 3C), a convergent feature that is shared with other burrowers such as *Hemisus* (headfirst [45]) and *Rhinophrynus dorsalis* (hind-feetfirst [46]). Increased anterior deployment of the pectoral girdle provides a “mechanical-coupling” with the body, thus providing increased surface area for attachment of pectoral musculature [45] and possibly increasing maneuverability of the forelimbs involved in forward movement [46]. This anterior placement of the pectoral girdle has been used as one of the prominent characters of headfirst burrowers (*Glyphoglossus molossus* [45]). Even though *Nasikabatrachus* uses its hind feet to initiate burrowing, it may move through the soil headfirst, especially when searching for food. A wedge-shaped skull with a thick epidermal knob at the tip of the pointed snout and forelimbs with well-ossified prepollical elements likely would facilitate forward movement underground, which would be useful for locating new food sources and refuges (as also noted for *Rhinophrynus dorsalis* [46]).

In larval *Nasikabatrachus*, endolymphatic sacs invade the extradural space of both the cranium and the vertebral column; they extend posteriorly to the sacrum. *Nasikabatrachus* possesses “type V” endolymphatic sacs, which “are large and lie in the extradural space of both the endocranial and vertebral cavities” [48]. They develop and expand as larval development progresses (Fig 4A–4F) but persist even in well-developed metamorphs (stages 45–46; Fig 4D). The sacs are made of aragonite, a form of calcium carbonate [22,48] and likely represent an important source of calcium for bone formation during and after metamorphosis.

Our study provides material for comparative analyses with other anurans as well as insights into the varied survival strategies of amphibians. The two highly specialized life history stages of *Nasikabatrachus*, clinging tadpoles and burrowing adults, display many peculiar features. This relic from the Jurassic era reminds us that extreme specialization can be an effective survival strategy over evolutionary time.

## Supporting Information

S1 Fig Cranial and postcranial ossification indices versus developmental (Gosner) stage in *Nasikabatrachus sahyadrensis*. (A) Skull. (B) Postcranial skeleton. In each comparison, ossification index equals the number of bones present at a given stage divided by the total number of bones present at the end of metamorphosis (skull, 11; postcranial skeleton, 79).
(JPG)

S1 Table External morphological measurements of *Nasikabatrachus sahyadrensis*. (XLSX)

S2 Table Cranial and postcranial bones of *Nasikabatrachus sahyadrensis* in different developmental stages. (XLSX)
