# Supplementary figures and images for: From Clinging to Digging: The Postembryonic Skeletal Ontogeny of the Indian Purple Frog, Nasikabatrachus sahyadrensis (Anura: Nasikabatrachidae)
Source: PLoS One. 2016 Mar 30;11(3):e0151114. doi: 10.1371/journal.pone.0151114 (PMC4814056; doi:10.1371/journal.pone.0151114)

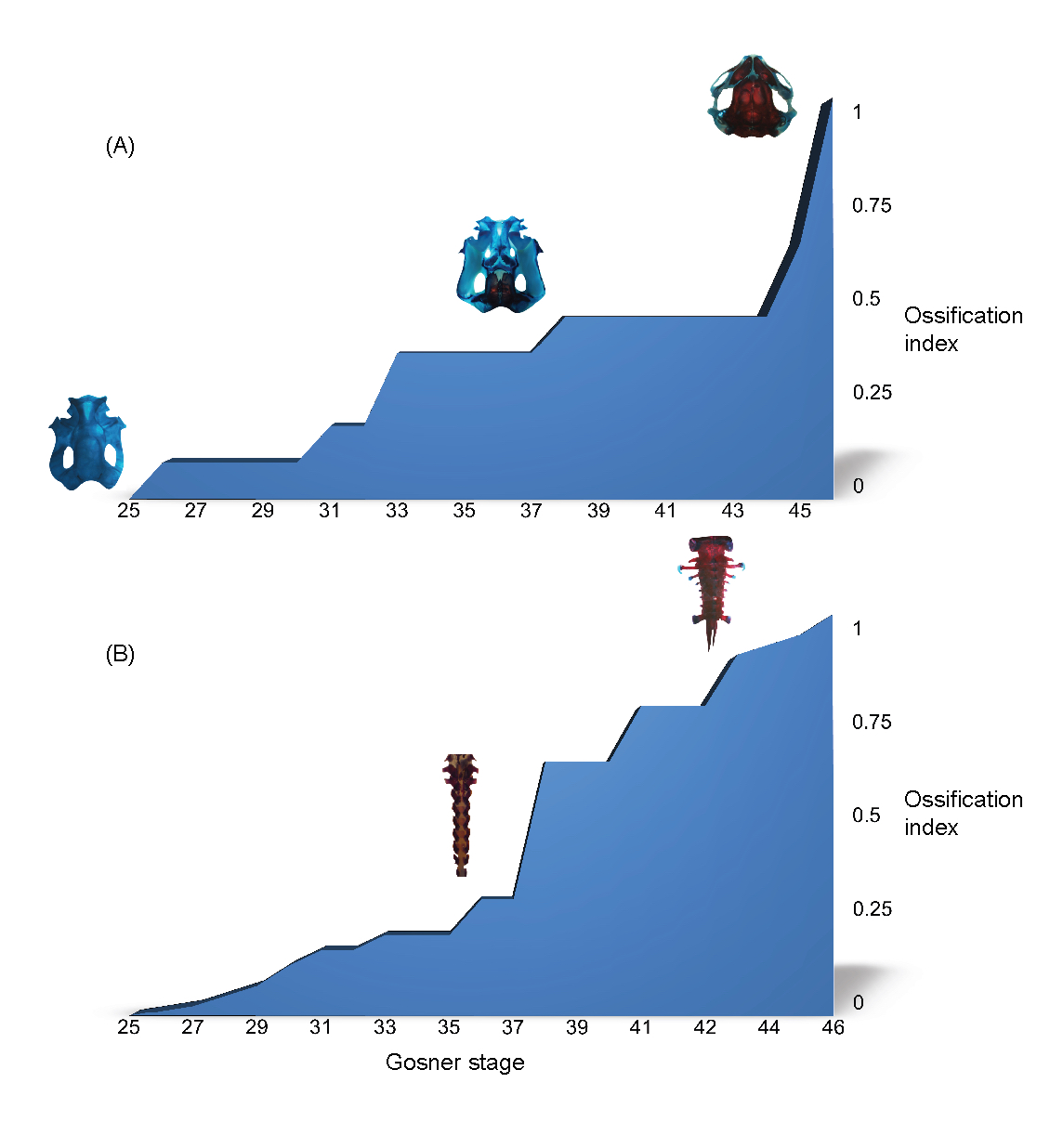

Supplement: S1 Fig — (A) Skull. (B) Postcranial skeleton. In each comparison, ossification index equals the number of bones present at a given stage divided by the total number of bones present at the end of metamorphosis (skull, 11; postcranial skeleton, 79). (JPG) [file pone.0151114.s001.jpg]
